# Pleomorphic Lobular Carcinoma of the Mammary Gland in Women and Female Dogs: A Comparative Clinical-Pathological and Immunophenotypic Analysis

**DOI:** 10.3390/vetsci12060587

**Published:** 2025-06-14

**Authors:** Evelyn Ane Oliveira, Lize Amanda Basaglia Borges, Thaynan Cunha Vieira, Bárbara Jaime dos Santos, Fernanda Rezende Souza, Karen Yumi Ribeiro Nakagaki, Cristiana Buzelin Nunes, Geovanni Dantas Cassali

**Affiliations:** 1 Department of General Pathology, Institute of Biological Sciences, Federal University of Minas Gerais, Av. Presidente Antônio Carlos, 6627, Belo Horizonte 31270-901, Brazil; evelyn.anee@gmail.com (E.A.O.); lizebborges@gmail.com (L.A.B.B.); thaynan07@gmail.com (T.C.V.); fersouza.vet@gmail.com (F.R.S.); karenyumi@ymail.com (K.Y.R.N.); 2 Department of Pathology and Forensic Medicine, Faculty of Medicine, Federal University of Minas Gerais, Av. Prof. Alfredo Balena, 190, Belo Horizonte 30130-100, Brazil; barbara.biomed@hotmail.com (B.J.d.S.); cristianabnunes@gmail.com (C.B.N.)

**Keywords:** canine, mammary, carcinoma, woman, breast, immunohistochemistry

## Abstract

Pleomorphic invasive lobular carcinoma (PILC or PLC) is a malignant breast tumor considered a rare variant of invasive lobular carcinoma in women, characterized morphologically by marked nuclear pleomorphism, with cells resembling plasmacytoid, histiocytoid, or apocrine cells. One of its defining features is the loss of E-cadherin expression. Considering the biological similarities between species and the limited data available for female dogs, this study aimed to characterize PLC in canines, with an emphasis on its histopathological and immunophenotypic features and its potential applicability as a comparative model. Histopathological analysis of PLC was performed alongside immunohistochemical evaluation using HER2, estrogen receptors (ER), progesterone receptors (PR), E-cadherin, and Ki-67 (cell proliferation indexing) markers. All canine PLCs tested positive for PR, with the majority being negative for ER, and all were negative for HER2 and E-cadherin. In contrast, in women, all cases were positive for ER, most were positive for PR, and all were negative for HER2. The Luminal B molecular subtype was the most frequent in dogs, whereas Luminal A and Luminal B subtypes showed equal prevalence in women. These findings reveal shared and distinct immunophenotypic features between species. The similarities and differences observed emphasize the relevance of the canine model for comparative oncology. Furthermore, the use of spontaneous tumors in immunocompetent dogs in this study strengthens the translational potential of the findings, thereby reinforcing the use of the canine model in breast cancer research and supporting its role within the One Medicine concept.

## Simple Summary

Pleomorphic lobular carcinoma is a rare and aggressive type of breast cancer in both women and female dogs. This study aimed to better understand this tumor by comparing cases from humans and dogs. Tissue samples were analyzed using traditional staining and immunohistochemical techniques to assess pathological and immunophenotypic features. The findings showed that, although the tumors in both species share many similarities in their appearance and behavior, there are important differences, particularly in how they express hormone receptors and proliferation rate. In dogs, these tumors showed a higher rate of cell growth and consistent positivity for the progesterone receptor, while in women, most tumors were positive for both estrogen and progesterone receptors. Both species showed a loss of a protein important for cell adhesion, which may relate to neoplastic cell dissemination. Recognizing these similarities and differences helps to better understand breast cancer and opens the door to using dogs as a model to study this disease. Despite the current limitations such as the lack of follow-up data and clinical staging, this study provides an important first step in recognizing pleomorphic lobular carcinoma as a comparable tumor in dogs. Future research should focus on clinical outcomes, distant metastasis, and tumor microenvironment to clarify prognostic factors and confirm the translational relevance of this spontaneous model in comparative oncology.

## 1. Introduction

Pleomorphic lobular carcinoma (PILC or PLC) is a rare variant of invasive lobular carcinoma (ILC), accounting for approximately 15% of ILC cases and 1% of all invasive breast carcinomas in women [1,2,3]. This subtype was first described in veterinary medicine in 2002 by Cassali et al., who identified histological similarities to human PLC, such as atypically dispersed cells with eccentric nuclei and, occasionally, abundant cytoplasm [4]. In humans, PLC shares a histopathological growth pattern with classic ILC, featuring single rows of discohesive cells arranged in one to two layers, forming the characteristic “Indian file” pattern [5]. However, PLC is distinguished by marked nuclear pleomorphism, with tumor cells displaying plasmacytoid, histiocytoid, or apocrine morphology [6]. A defining feature is the loss of E-cadherin expression, frequently accompanied by variations in the expression of estrogen and progesterone receptors (ER and PR), and, although often HER-2 negative, some cases demonstrate HER-2 amplification [1,2,7,8].

PLC in humans is associated with poorer survival compared to both classic ILC and invasive ductal carcinoma, due to adverse histopathological characteristics, larger tumor size, lymph node metastasis, and aggressive molecular profiles [1,9,10,11]. In veterinary medicine, there is currently no universally accepted classification that recognizes PLC as a distinct histological subtype in canine mammary tumors. This reflects, in part, the variability in diagnostic criteria adopted by research groups from different countries. Despite this, PLC has previously been reported in one cat [12] and five female dogs based on their marked morphological and immunohistochemical resemblance to the human counterpart [4,13,14,15,16].

To our knowledge, this is the first study to compare and characterize the clinicopathological and immunohistochemical features of PLC in 26 cases across both species. The objective of this study is to elucidate the existence and potential inclusion of PLC as a distinct entity in future veterinary classifications in female dogs, emphasizing its aggressive behavior and histological features that closely resemble those observed in women. Additionally, given the growing relevance of immuno-oncology in aggressive subtypes such as PLC, the spontaneous and immunocompetent nature of canine mammary tumors represents a valuable opportunity for comparative research and model development.

## 2. Materials and Methods

A retrospective study was performed by selecting cases based on patient reports, from which information such as diagnosis, age, tumor size, lymph node status, and type of surgery was extracted.

A total of 26 formalin-fixed paraffin-embedded canine samples diagnosed with pleomorphic lobular carcinoma between 2009 and 2020 were selected from the archives of two veterinary diagnostic laboratories located in the city of Belo Horizonte, Minas Gerais, Brazil—the Laboratory of Comparative Pathology (LPC) at the Institute of Biological Sciences, Federal University of Minas Gerais (ICB-UFMG), and Celulavet—Veterinary Diagnostic Center. Additionally, 26 human samples were obtained from the archives of the Mammary Pathology Laboratory, School of Medicine, UFMG, covering the years 2010 to 2020, with diagnoses of high-grade invasive lobular carcinoma and pleomorphic invasive lobular carcinoma. Histopathology: Histological sections of 4 µm were obtained from paraffin-embedded tumor blocks and processed using conventional techniques for hematoxylin and eosin (H&E) staining. The classification of canine tumors followed the criteria established by the Consensus for the Diagnosis, Prognosis, and Treatment of Canine Mammary Tumors [17], while human tumors were classified according to the World Health Organization (WHO) criteria [18]. Two cases of ILC with high histological grade were reviewed by a human pathologist with experience in breast pathology (CBN) and reclassified as pleomorphic invasive lobular carcinoma. Histological reclassification was based on the criteria established by the World Health Organization (WHO), which define PLC as retaining the characteristic growth pattern of classic ILC while exhibiting more pronounced cytologic atypia, marked nuclear pleomorphism, increased mitotic activity, and, in some cases, apocrine, histiocytoid, or signet ring cell differentiation. Photomicrographs were captured with an Olympus BX41 microscope, and images were digitized using the SPOT^®^ Insight Color software (version 3.4.5; Diagnostic Instruments Inc., Sterling Heights, MI, USA).

Histological Grading and Clinical Staging: Human and canine samples were evaluated and classified using the Nottingham grading system, which considers three criteria: tubule formation, nuclear pleomorphism, and mitotic index. For the analysis of clinical and histopathological parameters related to tumor progression, the TNM staging system was applied. In this system, (T) represents the size and extent of the primary tumor, (N) refers to the evaluation of regional lymph nodes for the presence or absence of metastases, and (M) denotes the presence or absence of distant metastases [19]. Tumor histological grade was determined by summing the scores, with classification into grade I (3–5 points), grade II (6–7 points), or grade III (8–9 points) [20].

Immunohistochemistry: Histological sections with a thickness of 4 µm were obtained from paraffin-embedded tissue blocks and mounted on gelatin-coated slides. After deparaffinization in xylene and alcohol, the sections were rehydrated using decreasing concentrations of alcohol. For canine samples, the Novolink Polymer Detection System Kit (Leica Biosystems, Newcastle, Tyne, UK) was utilized, whereas the Advance™ HRP Detection Kit (Dako North America, Carpinteria, CA, USA) was applied for human samples, following the manufacturers’ instructions. Antigen retrieval for ER, PR, Ki67, HER2, and E-cadherin was performed using pressurized moist heat (Pascal) for both groups. In canine samples, a citrate buffer solution at pH 6.0 was used, while EDTA at pH 9.0 (Dako Cytomation Target Retrieval Solution, Dako, Glostrup, Denmark) was employed for human samples. Slides were incubated with appropriate primary antibodies, with human slides incubated for 1 h at room temperature and canine slides incubated for 16 h (overnight) in a humid chamber at 4 °C. All of the antibodies used had been properly tested and had previously been used in other canine studies. Negative and positive controls were also employed to ensure the reliability of the reaction. Immunoreactivity was visualized using the chromogen 3,3′-diaminobenzidine (DAB Substrate System, Dako, Carpinteria, CA, USA) and counterstained with Mayer’s hematoxylin. Detailed information on the antibodies, including manufacturers, clones, and dilutions, is provided in Table 1 and Table 2.

Immunohistochemical evaluation: The Ki67 cell proliferation index was determined by manually counting the number of positive nuclei in a total of 1000 neoplastic cells from areas of optimal staining (“hot spots”), using manual image analysis via Image J software (v.2.3.0, National Institute of Health, Bethesda, MD, USA). A cutoff value of 20% was used to classify the proliferation index as high or low in dogs and discriminate between luminal A and luminal B subtype, as proposed by Nunes et al. (2018), who observed an association with lower survival rates [21], and 30% in humans, according to Nielsen et al. (2021) [22]. ER and PR expression were considered positive if more than 1% of neoplastic cells exhibited staining [23]. HER-2 expression was evaluated using the semi-quantitative scoring system established by the American Society of Clinical Oncology/College of American Pathologists [24]. Cases were classified as positive if strong, complete membranous staining was observed in more than 10% of neoplastic cells (3+). Based on immunohistochemical results, the cases were classified into five groups: Luminal A, Luminal B (HER-2 negative or positive), HER-2 overexpressed, and triple negative, as described in Table 3.

Statistical Analyses: Statistical analyses were performed using GraphPad Prism 8 software. The Mann–Whitney U test was applied to compare immunohistochemistry results between pleomorphic lobular carcinomas (PLCs) in humans and dogs. For data with a normal distribution, assessed via the Shapiro–Wilk test, the *t*-test was used. *p*-values less than 0.05 were considered statistically significant.

## 3. Results

The age of the dogs at the time of mammary gland surgery ranged from 4 to 12 years (mean 9.2 years; n = 22). In six cases, age information was unavailable in the clinical records. Among women, the age range was 42 to 77 years (mean 56.7 years; n = 4), with two cases missing this information. Mixed-breed dogs were the most frequently represented (30.8%; 8/26), followed by Labrador Retrievers (26.9%; 7/26). Poodles and Yorkshire Terriers each accounted for 7.7% (2/26), while German Shepherds, Australian Shepherds, Golden Retrievers, Lhasa Apsos, and Dachshunds each comprised 3.8% (1/26). The breed was not reported in two cases.

Regarding tumor size in dogs, 26.9% (7/26) were classified as T1, 30.8% (8/26) as T2, and 38.5% (10/26) as T3, with one case lacking size information. For women, 34.6% (9/26) were classified as T1, 38.5% (10/26) as T2, and 11.5% (3/26) as T3, with four cases missing tumor size data. The assessment of metastases in regional lymph nodes was performed in 50% (13/26) of the dogs, of which 69.2% (9/13) presented lymph node metastases at diagnosis. Among women, lymph node data were available in 61.5% (16/26) of cases, and 43.8% (7/16) had metastases in one or more regional lymph nodes.

**Figure 1:**
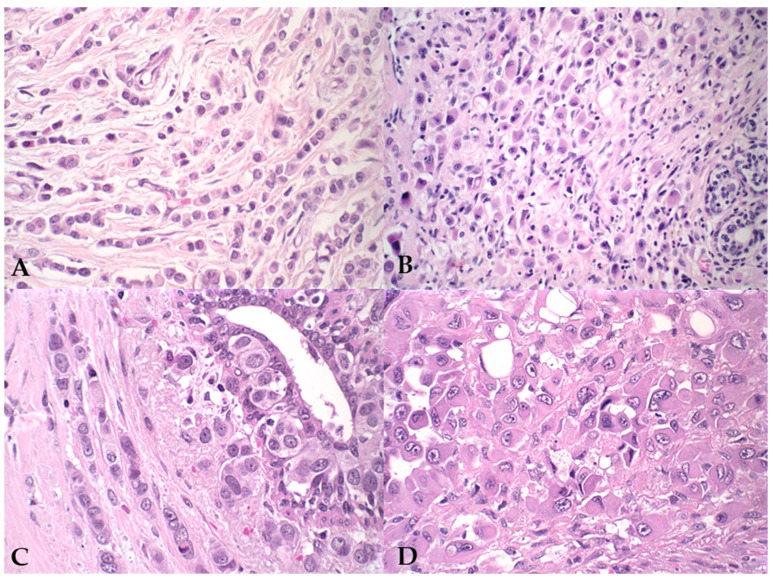
Photomicrographs illustrating pleomorphic lobular carcinoma in human and canine mammary glands. (**A**,**B**) Human PLC, H&E staining, 40× magnification; (**C**,**D**) Canine PLC, H&E staining, 60× magnification. The histopathological images show a discohesive arrangement of neoplastic cells with abundant cytoplasm, forming an Indian file pattern within the stromal tissue.

Both species exhibited histopathological characteristics indicative of poorly differentiated carcinomas, with abundant eosinophilic cytoplasm and varied cellular patterns, including plasmacytoid and signet-ring-like cells. The nuclei were markedly pleomorphic, with eccentric placement and prominent nucleoli. In certain regions, tumor cells were arranged in single-cell rows within the connective stroma, forming the characteristic “linear par” pattern (Figure 1).

Canine PLCs were associated with other tumor types in 20% of cases. Associations with micropapillary carcinoma were observed in 7.7% (2/26), areas of lobular carcinoma in situ (LCIS) in 7.7% (2/26), and solid carcinoma, mixed tumor carcinoma, tubular carcinoma, and carcinosarcoma in 3.8% (1/26) each. In women, 7.7% (2/26) presented LCIS-associated areas, while 3.8% (1/26) were associated with invasive ductal carcinoma areas.

Regarding histological grading in dogs, 65.4% (17/26) of the cases were classified as grade II, and 34.6% (9/26) as grade II. Similarly, in women, grade II was the most frequent, observed in 76.9% (20/26) of cases, followed by grade I in 15.4% (4/26), and grade III in 7.7% (2/26). The clinicopathological features are summarized in Table 4.

**Figure 2:**
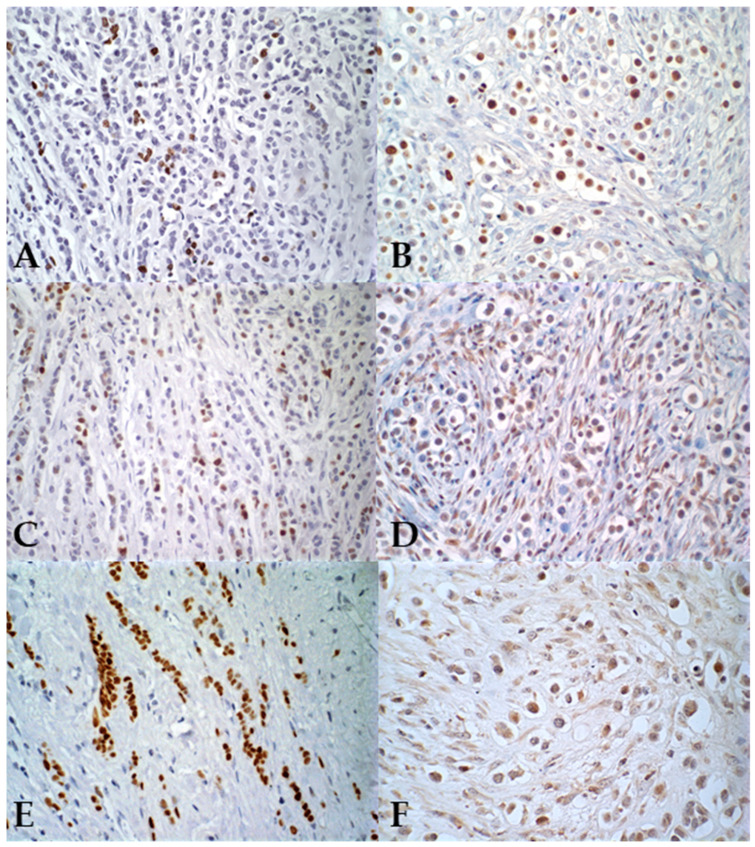
Photomicrographs illustrating features of pleomorphic lobular carcinoma in human (**A**,**C**,**E**) and canine (**B**,**D**,**F**) mammary glands, immunohistochemical (IHC) staining. (**A**,**B**) Nuclear staining with the proliferation marker Ki-67, IHC, 40× magnification; (**C**,**D**) nuclear staining of epithelial neoplastic cells for the progesterone receptor, IHC, 40× magnification; (**E**,**F**) nuclear staining of epithelial neoplastic cells for the estrogen receptor, IHC, 40× and 60× magnification, respectively.

Regarding hormonal markers, all female dogs tested positive for PR (100%–26/26), with the majority exhibiting between 50% and 75% of positive cells. For ER, only one female dog showed positivity (3.8%–1/26), while 96.2% (25/26) showed less than 1% of positive cells, classifying them as negative (Figure 2).

**Figure 3:**
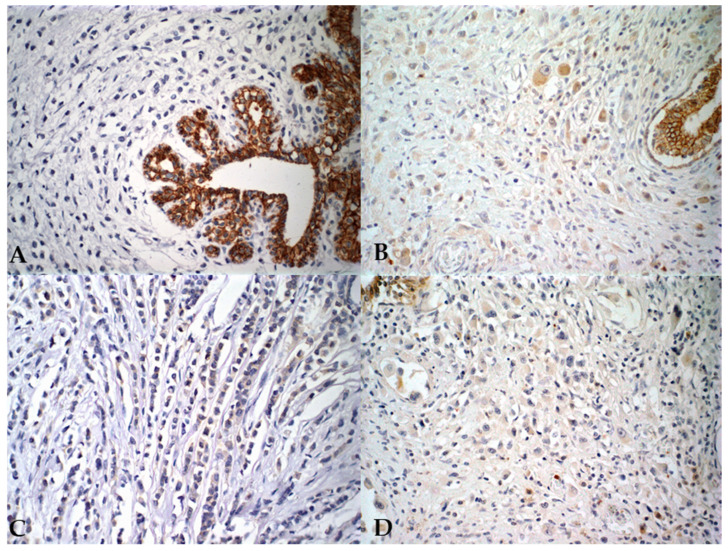
Photomicrographs illustrating characteristics of pleomorphic lobular carcinoma in human (**A**,**C**) and canine (**B**,**D**) mammary glands. (**A**,**B**) E-cadherin negativity in epithelial neoplastic cells from both species, with internal positive control visible in the lower left corner; immunohistochemical staining, 40× magnification. (**C**,**D**) Negative immunostaining for HER-2, IHC staining, 40× magnification.

In women, only one case presented negativity for PR (3.8%—1/26), and all women (100%—26/26) were positive for ER. The cellular proliferation index, measured by Ki67, ranged from 21.5% to 86.6% of positive cells in female dogs (average 58.7%), while in women it ranged from 3.9% to 38.5% (average 15%). These results are summarized in Table 5. In relation to HER-2 expression, all canine tumors showed negativity (100%—26/26). In women, one case (7.7%—2/26) was positive (3+), while the remaining cases were negative (92.3%—24/26) (Figure 3). Regarding the molecular subtype, all female dogs were classified as luminal B. Among women, 46.2% (12/26) were categorized as luminal A, 46.2% (12/26) as luminal B (HER-2^−^), and 7.7% (2/26) as luminal B (HER-2^+^).

## 4. Discussion

Studies have shown that mammary carcinomas in dogs and humans share various similarities, including malignant behavior, features related to aggressiveness, and morphological and cellular aspects [4,25,26].

In the present study, the immunophenotypic analysis revealed that in female dogs, pleomorphic lobular carcinoma was positive for PR in all cases. Regarding the ER, our findings contrast with previous studies in female dogs, which reported positivity for this receptor [4,15,16]. In humans, around 80% of PLC cases are positive for ER, and 60% for PR, suggesting that canine PLC may also exhibit both positivity and negativity for these markers [27,28]. This was further demonstrated when comparing the two groups statistically, where both showed *p* < 0.0001, indicating differences in hormonal receptor expression between the two species.

In contrast, our results for the PR align with findings in female dogs, which also showed positivity for this marker. Additionally, we observed negativity for HER-2, corroborating previous studies in female dogs. Statistically, there were no differences between human and canine PLC, suggesting that, for this marker, both species exhibit similar behavior [4,15,16]. Steroid hormones are known as potential risk factors for the development of neoplasms in both species [29,30,31,32]. Women with high levels of hormonal marker positivity may benefit from therapies with drugs such as tamoxifen, which could lead to increased survival [33]. In dogs, however, adverse effects have been associated with anti-estrogenic therapies [34].

Several thresholds for the proliferation index with Ki-67 have been described in veterinary medicine. Recent studies using a cutoff of 20% showed a significant relationship with the survival time of animals [35,36]. Other studies indicated that values above 20% are associated with regional lymph node metastases and tumors larger than 5 cm [37,38].

In this study, Ki-67 indices in female dogs were statistically higher than those observed in women, indicating a higher cellular proliferation rate in the canine species, highlighting the difference between the two groups. Studies indicate that Ki-67 indices in pleomorphic lobular carcinomas are high, in addition to showing a higher histological grade compared to other carcinoma subtypes [39,40,41]. In humans, a high Ki-67 index is associated with a poor prognosis, although there is a good response to chemotherapy due to the elevated cellular proliferation rate [42]. Similarly, in dogs, despite the association with worse prognosis, individuals presenting Ki-67 indices above 20% who underwent chemotherapy demonstrated improved survival outcomes [21,43].

PLC is characterized by the negativity of E-cadherin, which was confirmed in this study, as the invasive areas showed no positivity. In transition regions from in situ to invasive, weak and incomplete staining of tumor cells was observed, indicating the progressive loss of this adhesion molecule as the tumor advances [7,44,45]. The average age of animals in this study is consistent with that observed in women, reinforcing the dog as a comparative model for human cancer, as suggested by Lebeau (1953) [46] and Wang (2020) [47]. Tumor size, often discussed in canine and human literature, is an important prognostic factor. Tumors larger than 3 cm in dogs are considered worse prognosis and are considered an independent prognostic factor [48].

Regional lymph node metastasis is an important prognostic factor in both dogs and women, which can reduce survival, especially when associated with other prognostic indicators [49]. However, no information on distant metastasis was available for either species, limiting comprehensive clinical staging. Histological grade is an indicator of tumor aggressiveness, influencing disease-free interval and overall survival in both canine and human patients. In this study, pleomorphic lobular carcinomas (PLC) in both species exhibited moderate to high histological grades, which, along with high Ki-67 indices and loss of E-cadherin expression, supports the characterization of PLC as an aggressive neoplasm across species. Nevertheless, several limitations must be considered. The retrospective nature of the study and the use of archived samples resulted in incomplete clinical records, including missing data on age, tumor size, and lymph node status in some canine cases. Furthermore, the absence of follow-up information, such as overall survival, disease-free survival, or treatment outcomes, limited the possibility of correlating histopathological and immunohistochemical features with clinical behavior. The absence of data regarding therapeutic interventions also precluded discussions on potential implications for treatment across species. Therefore, future studies should incorporate molecular profiling, longitudinal clinical outcomes, and detailed follow-up to expand our understanding of PLC’s biological behavior and strengthen the translational relevance of canine models in comparative oncology.

## 5. Conclusions

Pleomorphic lobular carcinomas (PLCs) in female dogs and women exhibit similar histological and immunophenotypic features, supporting the view that these tumors represent comparable entities across species. Similar mechanisms of tumor progression appear to occur in both species, as indicated by alterations in cell adhesion markers during the transition from carcinoma in situ to invasive carcinoma. However, distinct biological behaviors were observed, particularly in hormone receptor expression and proliferative activity, suggesting species-specific tumor dynamics. Although differences in ER and PR expression were noted, the molecular mechanisms underlying these variations were not investigated. Histological grade and regional lymph node metastasis remain important indicators of prognosis in both species, significantly impacting survival rates and disease-free intervals.

Considering the limitations of this study, future research should prioritize longitudinal clinical data, molecular profiling, and immune characterization. Investigating the immune microenvironment of canine PLCs is essential to elucidate the role of host immune responses in tumor development and progression. Integrating molecular and functional profiling of immune components may provide further insight into tumor biology and improve the utility of the canine model in comparative breast cancer research. These are essential to validate prognostic correlations, refine comparative models, and enhance the translational relevance of canine PLCs in breast cancer research.

In conclusion, while PLCs in female dogs and women share fundamental pathological characteristics, species-specific differences may influence tumor behavior and prognosis. These findings highlight the importance of comparative pathology in advancing the understanding of tumor biology and improving diagnostic and prognostic approaches across species.

## Figures and Tables

**Table 1:** Primary antibodies used for human mammary tumors, including manufacturers, clones, and dilutions.

Antibody	Manufacturer	Clone	Dilutions
PR	Dako	HPRA2	1:50
ER	Dako	1 D5	1:50
KI67	Dako	Mib-1	1:50
E-Cadherin	Zymed (San Francisco, CA, USA)	4 A2 C7	1:50
Her-2	Dako	Polyclonal	1:200

**Table 2:** Primary antibodies used for canine mammary tumors, including manufacturers, clones, and dilutions.

Antibody	Manufacturer	Clone	Dilutions
PR	Dako	SP2	1:200
ER	Dako	EP1	Ready to use
KI67	Dako	Mib-1	1:180
E-Cadherin	Bio SB inc. (Goleta, CA, USA)	EP700 Y	1:180
Her-2	Dako	Polyclonal	1:180

**Table 3:** Criteria for determining molecular subtypes.

	Luminal A	Luminal B		Her2 Overexpressed	Triple Negative
		Her2-Negative	Her2-Positive		
RE and/or RP	+	+	+	–	–
HER-2	–	–	+	+	–
Ki67	<20%	≥20%	AS *	AS	AS

* AS: Any score.

**Table 4:** Clinical and histopathological characteristics of canine PLC and human PLC.

	Canine PLC	Human PLC	*p*-Value
Mean age (years)	9.2	56.7	
Tumor Size	% (n)		
<3 cm	26.9 (7)	-	
3 to 5 cm	30.8 (8)	-	
>5 cm	38.5 (10)	-	
Not available	3.8 (1)	-	
Tumor Size	% (n)		
<2 cm	-	35 (9)	
2 to 5 cm	-	38 (11)	
>10 cm	-	12 (3)	
Not available	-	15 (4)	
Breed	**%** (n)		
Mixed breed	30.8 (8)		
Labrador	26.6 (7)		
Histological grade	% (n)		0.0128
I	0	15.4 (4)	
II	65.4 (17)	76.9 (20)	
III	34.6 (9)	7.7 (2)	
Lobular Carcinoma In situ	% (n)		1.0
Absent	92 (24)	96 (24)	
Present	8 (2)	4 (1)	
Lymph Node Metastasis	% (n)		1.0
Absent	35 (9)	27 (7)	
Present	15 (4)	35 (9)	
Not available	50 (13)	38 (10)	

**Table 5:** Comparison of immunohistochemical markers in canine and human pleomorphic lobular carcinomas.

	Canine PLC	Human PLC	*p*-Value
PR			<0.0001
	100 (26/26)	96.2 (25/26)	
	0	3.8 (1/26)	
ER			<0.0001
	3.8 (1/26)	100 (26/26)	
	96.2 (25/26)	0	
***HER-2***			0.49
	100 (26/26)	92.3 (24/26)	
	0	7.7 (2/26)	
KI67			<0.0001
Mean ± SD (Max–Min) %	58.65 ± 18.81 (88.8–21.5)	15.73 ± 8.48 (38.0–4.0)	

## Data Availability

The original contributions presented in the study are included in the article; further inquiries can be directed to the corresponding author.

## Abbreviations

The following abbreviations are used in this manuscript:

PILC or PLC	Pleomorphic invasive lobular carcinoma
ILC	Invasive lobular carcinoma
ER	Estrogen receptor
PR	Progesterone receptor
HER-2	Human epidermal growth factor receptor 2
LPC	Laboratory of Comparative Pathology
ICB–UFMG	Institute of Biological Sciences–Federal University of Minas Gerais
WHO	World Health Organization
TNM	Size; regional lymph node; distant metastasis.
AS	Any score
